# An image-based screen for secreted proteins involved in breast cancer G0 cell cycle arrest

**DOI:** 10.1038/s41597-024-03697-z

**Published:** 2024-08-10

**Authors:** William A. Weston, Jordan A. Holt, Anna J. Wiecek, James Pilling, Lovisa Holmberg Schiavone, David M. Smith, Maria Secrier, Alexis R. Barr

**Affiliations:** 1 MRC Laboratory of Medical Sciences, Imperial College London, Du Cane Road, London, W12 0HS UK; 2 Institute of Clinical Sciences, Imperial College London, Du Cane Road, London, W12 0HS UK; 3 UCL Genetics Institute, Department of Genetics, Evolution and Environment, University College London, London, UK; 4 Discovery Biology, Discovery Sciences, R&D, AstraZeneca, Cambridge, CB2 0AA UK; 5 Discovery Biology, Discovery Sciences, R&D, AstraZeneca, Gothenburg, Sweden; 6 Emerging Innovation Unit, Discovery Sciences, R&D, AstraZeneca, Cambridge, CB2 0AA UK

**Keywords:** Breast cancer, Cell-cycle exit, Cellular imaging, Extracellular signalling molecules

## Abstract

Secreted proteins regulate the balance between cellular proliferation and G0 arrest and therefore play important roles in tumour dormancy. Tumour dormancy presents a significant clinical challenge for breast cancer patients, where non-proliferating, G0-arrested cancer cells remain at metastatic sites, below the level of clinical detection, some of which can re-enter proliferation and drive tumour relapse. Knowing which secreted proteins can regulate entry into and exit from G0 allows us to manipulate their signalling to prevent tumour relapse. To identify novel secreted proteins that can promote breast cancer G0 arrest, we performed a secretome-wide, image-based screen for proteins that increase the fraction of cells in G0 arrest. From a secretome library of 1282 purified proteins, we identified 29 candidates that promote G0 arrest in non-transformed and transformed breast epithelial cells. The assay we have developed can be adapted for use in other perturbation screens in other cell types. All datasets have been made available for re-analysis and our candidate proteins are presented for alternative bioinformatic refinement or further experimental follow up.

## Background

Proliferating cells can exit the cell cycle into a state of G0 arrest. The term G0 arrest is most broadly used to describe any non-proliferating cell, including the reversible arrest state of quiescence and the irreversible arrest state of senescence^1,2^, and cells that exit the cell cycle from either G1 or G2^3–6^. Tight control of cell proliferation is essential for healthy tissue development and homeostasis, and aberrant proliferation drives tumorigenesis^7,8^. Tumour dormancy describes residual tumour cells which can evade treatment and immune clearance to survive below detectable levels in patients, often at metastatic sites, before re-entering a proliferative state and driving tumour relapse^9,10^. Tumour cells that reawaken can be said to be in quiescence since they are able to return to proliferative cell cycles. Recurrent tumours can appear as long as 25 years after primary treatment in oestrogen-receptor positive (ER+) breast cancer and are frequently metastatic, with more aggressive therapy resistant phenotypes^11,12^. Given the lack of treatment options for tumour dormancy, recurrent metastatic disease remains a significant clinical challenge to cancer patients^13–15^. One goal to prevent dormant tumour cell reawakening is to maintain an indefinite G0 arrest in cancer cells.

Transitions into and out of G0 arrest are controlled by intrinsic and extrinsic cues which converge on the activity of cyclin-CDK complexes to regulate cell cycle gene expression^16,17^. If cyclin-CDK activity reaches, or is maintained at, a minimum threshold, then cells will proliferate. Below that threshold, and cells will remain in, or enter, G0 arrest. We and others have previously shown that intrinsic DNA damage, or replication stress, occurring during normal proliferation, can promote a “spontaneous” p53- and p21-dependent G0 arrest after mitotic exit^18–22^. Secreted proteins, including growth factors, cytokines, surface receptors, and extracellular matrix (ECM) components, are extrinsic factors that play a key role in G0 arrest-proliferation decisions, both in healthy tissue and in regulation of the tumour microenvironment^23–28^. For example, secretion of thrombospondin-1 in breast cancer vasculature^29,30^, or osteopontin and TGFb2 in the bone marrow of leukaemia patients, have been shown to induce G0 arrest in tumour cells^31,32^. TGFb1 has also been shown to induce G0 arrest in squamous cell carcinoma cells^33^, while IFN-1 and IFN-γ can induce G0 arrest in lymphoblasts or hepatocytes respectively^34,35^.

The secretome comprises approximately 13% of human protein-coding genes, yet the function of most secreted proteins remains poorly characterised^36,37^. Despite this, ~70% of clinically approved treatments use or target secreted proteins, or cell surface associated membrane proteins^38^. Considering their significant functional relevance in regulating G0 arrest, and apparent translational potential, secreted proteins present a valuable opportunity to target tumour dormancy processes. However, a systematic investigation of the secretome and its role in cancer cell G0 arrest has not been performed. Therefore, to identify novel secreted proteins that can promote G0 arrest in breast cancer cells, we developed a high-throughput, secretome-wide, image-based protein screen. Our screen used a purified secreted protein library (1282 proteins), generated by AstraZeneca in collaboration with the Royal Institute of Technology (KTH) in Stockholm^39,40^, but our methodology can be applied to any perturbation screen to identify proteins that regulate proliferation-G0 transitions^37^. Here, we use the term G0 arrest in its broadest sense, encompassing any cell in a population that is outside the proliferative cycle, in a given timeframe. Additional downstream analyses would be needed beyond the screen to identify the nature of G0 arrest in response to any given protein. Portions of this secretome library have been previously used in screens to identify a role for FGF16 in stimulating proliferation of cardiac progenitor cells^39^, to uncover roles for GDF-7, PAP, IFNα-7 in stabilising Regulatory T-cell (Treg) phenotypes^41^ and revealing the impact of FGFs 1, 4, 9 and 18 on pancreatic Β-cell differentiation^42^.

**Fig. 1:**
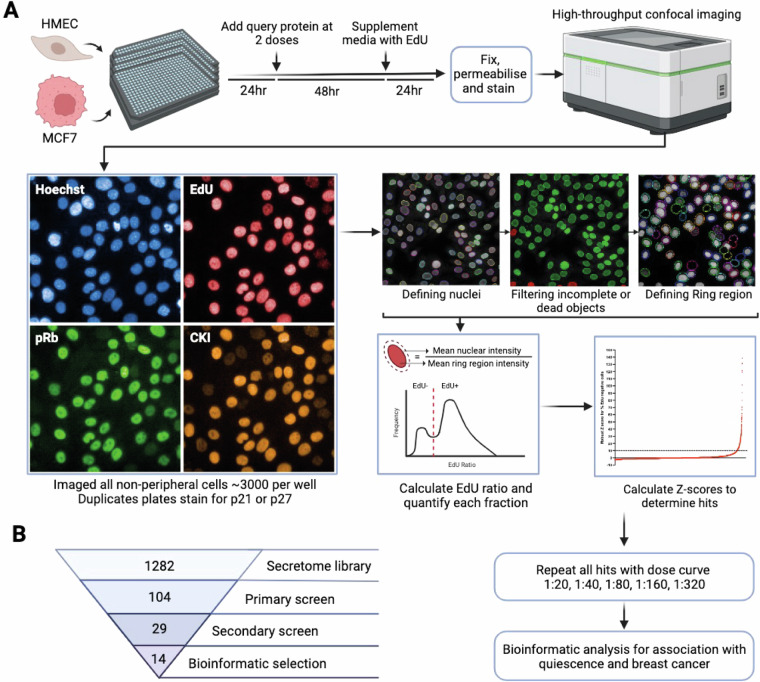
Experimental workflow. (**a**) The screen methodology is depicted starting with the primary screen, including staining, imaging, analysis and hit ranking, followed by a secondary screen of hits and bioinformatic shortlisting. Representative images of each stain are depicted, with CKI (CDK inhibitors) representing p21 or p27. The image analysis pipeline is represented by screenshots from Harmony (PerkinElmer) software. (**b**) Flow chart depicting how the secretome library was refined at each stage to a final list of proteins which induce G0 arrest.

Figure 1 shows the workflow for the screen. Briefly, the secretome protein library was added to either telomerase-immortalised, non-transformed human mammary epithelial cells (hTERT-HMEC) or ER + breast cancer cells (MCF7) for three days, after which cells were fixed and imaged for cell cycle markers. Using both hTERT-HMEC and MCF7 cells allows us to potentially identify common versus context-specific hits, or where G0 arrest pathways may have been lost in breast cancer. A number of cell cycle markers were used in the screen to identify G0 arrest phenotypes and characterise cell states. The thymidine nucleotide analogue 5-Ethynyl-2′-deoxyuridine (EdU) was added for the last 24 h of the screen. EdU is incorporated into proliferating cells during DNA replication (S-phase)^43^. Therefore, any EdU negative (EdU-) cells are non-proliferative and defined as G0 arrested. We used an antibody recognising phospho-S807/S811 Rb (retinoblastoma) protein. Rb is phosphorylated by cyclin-CDK complexes and marks commitment to proliferation^44–46^ and therefore P-Rb labels actively cycling cells. Because p53 wild-type cells can enter a spontaneous G0 arrest downstream of intrinsic DNA damage, these cell lines always display a significant fraction of EdU and P-Rb negative cells^18–20,22^. We immunostained cells for p21 and p27, both CDK inhibitors (CKIs) that can block cell cycle entry. Finally, Hoechst staining was used as a nuclear marker for segmentation and cell counting. We found EdU staining to be the most robust and therefore the most useful metric for quantification of G0 arrested cells and subsequent hit classification, while P-Rb, p21 and p27 staining provide more detailed information on the cell state at time of fixation (Fig. 1a).

**Fig. 2:**
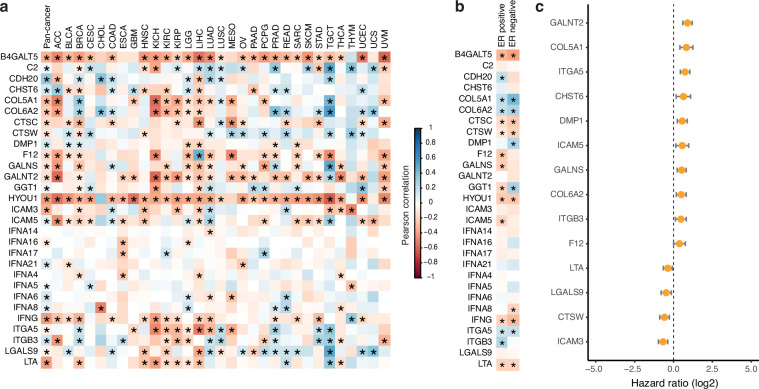
Bioinformatic analyses of hits. (**a**) Correlations between the G0 arrest score per tumour and the expression of selected secretome genes (encoding proteins that were hits in the screen) within the same tumour, calculated pan-cancer (first column) or by cancer type. Abbreviations correspond to different cancer types as defined by TCGA. Positive values (blue) indicate a positive correlation between G0 arrest levels and the expression of the respective gene, negative values (red) indicate an inverse relationship. Significant correlations (Pearson p < 0.05) are marked with an asterisk. (**b**) Correlations between the G0 arrest score and the expression of selected secretome genes across ER positive and ER negative breast cancers. Colour gradient and asterisk annotations as in (a). (**c**) Secretome genes with prognostic value in TCGA. The results from Cox proportional hazards analyses for overall survival across all cancers are shown, after adjustment for cancer type and stage. Samples with gene expression in the upper quartile were compared with those whose expression is in the lower quartile for the same gene. Positive values indicate that cases with high expression of the respective gene present worse survival.

**Fig. 3:**
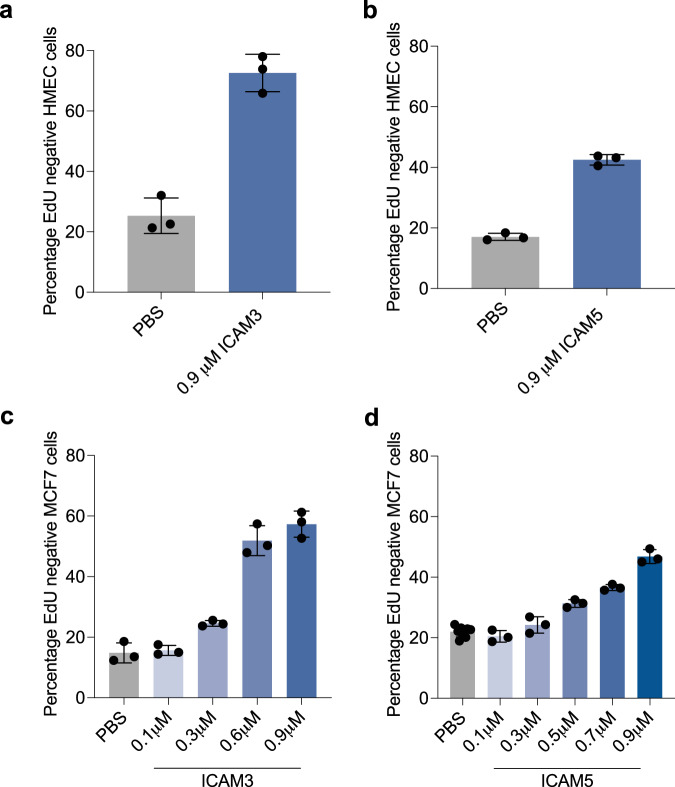
ICAM3 and ICAM5 promote G0 arrest in hTERT-HMEC and MCF7 cells. Graphs show commercial protein supplies of ICAM3 (**a**) or ICAM5 (**b**) addition to hTERT-HMEC cells. Mean +/− stdev of three technical repeats is shown. Graphs show dose curves of commercial protein supplies of ICAM3 (**c**) or ICAM5 (**d**) addition to MCF7 cells. Mean + /− stdev of three technical repeats is shown (except PBS controls for ICAM5 were nine technical repeats are shown).

Although our screen in theory could identify either increases in proliferation or in G0 arrest, we did not identify any secreted proteins that increased proliferation. This is perhaps not too surprising given that we screened immortalised cell lines growing in full growth media. Although these cell lines do contain a “spontaneous” G0 fraction, this spontaneous arrest has been shown, by us and others, to be p21-dependent, downstream of intrinsic replication stress^18–20,22^. Therefore, it is unlikely that a secreted protein would be able to reduce or overcome this spontaneous arrest and increase proliferation in this assay. Therefore, our screen is mainly useful for identifying proteins that increase G0 arrest. G0 arrest-inducing hits were rescreened in a secondary screen, with a dose curve of five concentrations (two-fold dilutions, ranging from 0.71-0.0066 μM). Remaining candidates were refined based on their correlation with G0 arrest penetrance^47^ across cancer types, and specifically in breast cancer, and association with cancer prognosis in publicly available datasets (Figs. 1b, 2). Here, G0 arrest penetrance refers to a validated score that can identify the relative fraction of G0 arrested cells in a tumour based on mRNA expression of 139 genes. One caveat is that all of these analyses are on primary tumours and therefore do not take into account the expression of these proteins in the metastatic niche where dormancy is likely to be more prevalent. Furthermore, the bulk nature of RNA-seq tumour profiles may confound some of the associations identified, as signals across all tumour cells are averaged (despite correction for immune/stromal content^47^). For instance, strong signals inducing G0 arrest in only one part of the tumour might be missed and could even show up as negatively correlated in extreme cases where most of the tumour is highly proliferative. Even so, these later steps of our screen can provide a more tailored selection of novel proteins with a possible role in breast cancer dormancy. To give us more confidence in the ability of our screen to identify meaningful hits, we also ordered purified proteins from commercial suppliers for ICAM3 and ICAM5 and showed that these also induce G0 arrest in HMEC cells (Fig. 3a,b). ICAM3 and ICAM5 were not identified as hits in MCF7 cells in our primary screen. However, we screened MCF7 cells with higher doses of the commercial proteins than those used in the primary screen and showed that ICAM3 and ICAM5 can promote G0 arrest in MCF7 at higher concentrations (0.45 and 0.3 μM, respectively; Fig. 3c,d).

We present here image data from our primary screen, with quantification of average nuclear intensities of Hoechst, EdU, p21, p27 and P-Rb, as well as features describing cell number, nuclear size, and nuclear shape for reanalysis^48^. We have included corresponding data from our validation screen based on G0 arrest induction^48^, and links to the datasets used for bioinformatic hit selection.

## Methods

### Cell Culture

MCF7 human adenocarcinoma cells (ATCC) were maintained in Dulbecco’s Modified Eagle Medium (DMEM from Gibco) supplemented with 10% FBS (Sigma) and 1% Penicillin-Streptomycin (Gibco). The hTERT-HMEC cells were a gift from Steve Elledge (Harvard) and were maintained in Mammary Epithelial Cell Basal Medium (PromoCell) with Mammary Epithelial Cell GM Supplement pack (PromoCell) and 1% P/S (Gibco). Both cell lines were grown at 37 °C and supplemented with 5% CO_2_ in humidified incubators. All cultures were maintained in T75 falcon flasks at a passage between P6 and P12 and consistently tested negative for mycoplasma contamination.

### Primary protein screen

A library of 1282 secretome proteins was provided by AstraZeneca in collaboration with the Royal Institute of Technology (KTH) in Stockholm. Access to this library is possible through AstraZeneca’s Open Innovation programme (https://openinnovation.astrazeneca.com/). Each protein in the screen was tested at two concentrations in hTERT-HMEC and MCF7 cells, at a single timepoint, in duplicate. The primary screen was performed at AstraZeneca’s labs in Cambridge.

#### Screening reagents

- Recombinant TGFb-1 protein (active) (Abcam, cat # ab50036), in 10 mM citric acid.
- Recombinant TGFb-2 protein (Abcam cat # ab84070), in PBS.
- Palbociclib hydrochloride (LKT Cambridge biosciences, cat # P0244) in DMSO.
- Click-iT EdU Cell Proliferation Kit for Imaging, (Invitrogen, cat # C10340 C) with AlexaFluor 647 dye.
- 8% methanol free formaldehyde in PBS (16% pre-made solution from Thermoscientific cat # 28908), final concentration 4%.
- PBS 1X pH 7.4 (Gibco, cat # 10010-023).
- Triton-X-100 (Sigma, cat # T9284), 0.5% in PBS.
- Bovine Serum Albumin (BSA)(Sigma), 2% in PBS, filtered.
- Rabbit monoclonal phospho-Rb S807/S811 antibody (CST, cat #9308) 0.5 μgml^−1^.
- Mouse monoclonal p21 antibody (Invitrogen, cat # MA5-14949) 1 μgml^−1^.
- Mouse monoclonal p27 antibody (BD, cat # 610241) 2 μgml^−1^.
- AlexaFluor 488 Goat anti Rabbit IgG antibody (Invitrogen # A11008) 1 μgml^−1^.
- AlexaFluor 568 Goat anti Mouse IgG antibody (Invitrogen # A11004) 1 μgml^−1^.
- Hoechst H33258 (Sigma) 1 μgml^−1^.

#### Day 0: Plating cells

Cell lines were cultured in T75 flasks (Falcon) to 70-80% confluency before seeding. MCF7 and hTERT-HMEC cells were seeded at a density of 1,000 cells per well in 30 μL of their normal culture media into black, optically clear bottom, 384 well Cell Carrier plates (PerkinElmer). Cells were left to attach overnight.

#### Day 1: Addition of secretome proteins

Purified proteins were diluted from their stock concentrations 2- and 20-fold in PBS, and 10 μL of each concentration was dispensed onto cells, to give a final volume of 40 uL per well, all using the Agilent Bravo. Each plate included neutral and positive controls; TGFb1 and TGFb2 both at a final concentration of 30 ng/mL, Palbociclib at a final concentration of 1 μM, and PBS. Proteins and controls were left to incubate with cells for 72 hr prior to fixation.

#### Day 3: Addition of EdU

10 μL of 50 μM EdU, diluted in cell media, was added to each well (final concentration 10 μM). Cells were incubated in EdU for 24 hr.

#### Day 4: Fixation and immunostaining

Fixation and immunostaining were carried out using an automated multidrop combi reagent dispenser and Biotek washer. Cells were washed three times in PBS between all steps, using the Biotek washer. Incubations were at room temperature, unless stated otherwise. Plates were fixed by adding 50 μL of 8% paraformaldehyde (PFA)/PBS on top of the media in the wells (4% final concentration) for 15 minutes, then permeabilised in 30 μL of 0.5% TritonX100/PBS for 15 minutes. EdU uptake by cells was visualised by Click-iT chemistry, according to manufacturer’s protocol using an Alexa-647 specific fluorophore. Next, cells were blocked in 30 μL of 2% BSA (filtered 0.2 μm)/PBS solution for 30 minutes, then 30 μL of primary antibodies in block solution were incubated at 4 °C overnight. Anti-phospho-Rb was added to both technical replicates and either anti-p21 or anti-p27 was added to a single screen replicate.

#### Day 5: Immunostaining and imaging

Fluorescently (Alexa)-labelled secondary antibodies were applied simultaneously for 1 hr in blocking solution and kept in the dark. Cells were stained for 15 minutes using 30 μL of 1 μM Hoechst H33258 in PBS for 15 minutes. Cells were washed and left in PBS for imaging. Plates were sealed using adhesive PCR sealing foil sheets (ThermoScientific) and kept at 4 °C. All plates were imaged using a CV7000 (Yokagawa) high content microscope in confocal mode (pinhole size of 50 μm). Four fields of view were taken per well using a 10x air objective, NA 0.4. On average over 1000 cells were quantified per well. Samples images from the primary screen^48^ are shown in 4a.

### Automated image analysis

All image analysis was performed using Columbus or Harmomy software (PerkinElmer). The custom image analysis method is described below.

#### Image analysis: Quiescent fraction determination

**Fig. 4:**
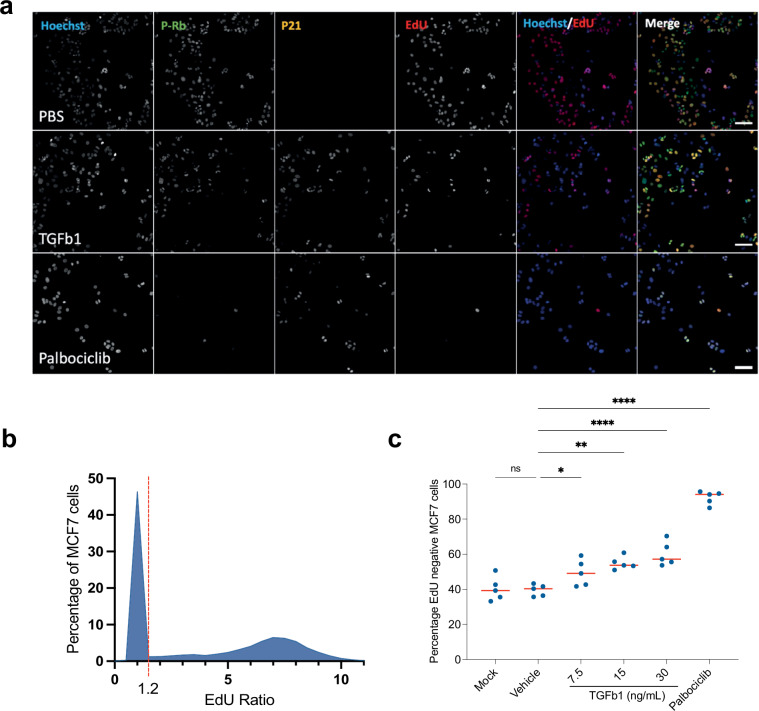
Positive controls for the screen that increase G0 arrest. (**a**) Representative images of screening controls in MCF7 cells - neutral (PBS), and positive controls for G0 arrest - TGFb1 and Palbociclib. In merged images Hoechst is shown in blue, P-Rb in green, p21 in yellow and EdU staining in red. Scale bar = 50 μM. (**b**) Frequency distribution of single cell data showing the EdU ratio. Dashed red line represents the cut off between EdU negative (−) and EdU positive cells. (**c**) Quantification of EdU negative (G0 arrested) cells. Graphs showing the percentage of cells per well which are EdU-. Red line represents mean. Mock = PBS treatment and Vehicle = DMSO. One-way ANOVA was used for statistical significance. ****p < 0.0001, ***p < 0.001, **p < 0.01, *p < 0.05, ns = not significant.

Nuclei were detected and segmented using Hoechst H33258 staining. Specifically, nuclei were detected using the Find Nuclei building block, channel marker BP445/45 (Hoechst) and using method ‘B’. Parameters used were: Common threshold 0.4, Area > 30 μm^2^ (excludes dead cells and debris), Splitting coefficient = 7, Individual threshold = 0.4 and Contrast > 0.1. Nuclei were further filtered to remove detected objects that were touching the edge of the image field of view using the ‘Select Population’ building block and the standard ‘Remove Border Objects’ method. The same method worked well for both cell lines and was used for all conditions. A ring region surrounding the nucleus was defined for each nucleus, ranging from 2-6μm outside the external edge of each nucleus, and the mean EdU intensity was measured within nuclear and nuclear ring regions. The EdU ratio was calculated for each nucleus by dividing the mean nuclear intensity by the mean ring region intensity, as a way of background normalisation. EdU ratio values were plotted in a frequency distribution that showed two clear populations (Fig. 4b). A ratio threshold of 1.2 was set between the two populations, meaning cells with an EdU ratio above 1.2 were classed as EdU+ (cycling) and cells with an EdU ratio below 1.2 were EdU- (G0 arrested, Fig. 4c). The percentage of EdU negative nuclei per well was then calculated to determine the G0 fraction. Although we excluded dead cells from the analysis, visual inspection of the wells and analysis of nuclear number (Supplementary Table 1) revealed that no proteins induced significant cell death (i.e. no protein addition gives nuclear number less than Palbociclib-induced cell cycle arrest). We noted that PBS addition in HMEC cells lead to lower nuclei counts in some wells compared to blank control wells which may be due to dilution of the media. However, importantly, PBS addition did not affect the fraction of EdU negative cells.

#### Additional feature extraction

Segmented nuclei were also counted in each well and their size and shape were calculated. The nuclear intensity of P-Rb (high is more proliferative, low is more G0 arrested) was quantified in all cells in all wells, and the nuclear intensity of p21 and p27 was quantified from the corresponding wells. All features have been recorded as the average value across all single cells for a given well (Supplementary Table 1).

### Hit determination

Data analysis was performed using the Genedata Screener platform (Genedata Ag). Each well was assigned a robust Z score based on the size of its G0 fraction. Each well was compared to the average of all wells in the screen to determine the strength of the effect that protein had on the G0 fraction. The Z score threshold for a significant increase of the G0 population was determined based on the distribution of hits and to include all positive controls and exclude neutral controls. Hits that increased the G0 fraction were defined as robust Z scores of >+3 in MCF7 and >+10 in hTERT-HMECs. To be classed as a hit, both technical repeats of a given protein at the same dose must have scored above the threshold in that cell line. Supplementary Table 1 shows the Z scores for all hits in each cell line, and Supplementary Table 2 shows a summary of hits that were hits at both concentrations of protein used in the screen. As expected, there was no evidence of hits decreasing the G0 fraction, so the remaining screen focused on proteins which increased the G0 fraction.

### Secondary screen (Hit validation)

All successful hits from the primary screen in either cell line (104 proteins, 83 from hTERT-HMEC, 21 from MCF7, Supplementary Table 2) were re-screened using an almost identical methodology and controls, though some modifications were made. Of note, while secondary screens were performed in the same cell stocks as the primary screen, secondary screens were performed in the labs at the MRC LMS which may account for differences in the fraction of EdU negative cells identified compared to the primary screen (Supplementary Table 1 versus Supplementary Tables 3, 4). However, despite these differences, each screen was compared to controls done on the same day to control for any differences in population growth. Due to limited protein availability, the 21 MCF7 hits were re-screened across 5 doses in both cell lines (Supplementary Table 3), whereas the 83 hits from hTERT-HMEC were only re-screened in hTERT-HMEC cells (Supplementary Table 4). In the secondary screen, on day 1, each protein was diluted in PBS from their stock concentrations (1:20, 1:40, 1:80, 1:160 and 1:320) using the automated liquid handler Opentrons OT to form a dose curve which was dispensed onto cells manually. The Palbociclib control was also titrated (1, 0.5, 0.25, 0.125, 0.0625 μM) while the PBS amount added remained constant. The remaining workflow remained unchanged, however, washing was performed with a Biotek 50TS microplate washer, widefield imaging was performed using an Operetta CLS (PerkinElmer) with a 20X objective NA 0.8, and an identical image analysis method was conducted using Harmony software (PerkinElmer). Hits were determined using the same parameters as our primary screen and are listed in (Supplementary Table 5).

Purified ICAM3 and ICAM5 were purchased from Abcam (ab276251 and ab276709, respectively) and were diluted in PBS. Cell treatment was as in the secondary screen.

### Bioinformatic screen

To cross reference which proteins in the screen are linked to breast cancer prognosis we explored the relation between their expression, G0 arrest and overall survival across multiple solid cancers from The Cancer Genome Atlas (TCGA). RNA-sequencing (FPKM normalised) and clinical data were downloaded using the *TCGABiolinks* R package^49^ for TCGA primary tumour samples across 31 solid cancer types. All expression data was log-transformed for downstream analysis. G0 arrest levels per bulk tumour sample were calculated as in^47^, based on a combined z-score of genes that are upregulated and downregulated, respectively, during G0 arrest.

#### G0 arrest associations

We calculated the Pearson correlation between the expression of each of the 1282 genes from the screen and the G0 arrest score calculated for each tumour sample, both pan-cancer as well as for individual cancer types. The genes most frequently correlated (either positively or negatively) with G0 arrest are shown in Fig. 2a, and thus represent the more likely candidates to contribute to G0 arrest in cancer. The associations appeared consistent regardless of the ER status in breast cancer (Fig. 2b).

#### Survival associations

Multivariate Cox Proportional Hazards analysis was carried out using the *coxph* function from the *survival* R package. Samples in the upper and lower quartile of expression for individual genes were compared, while adjusting for cancer type and stage. Genes with significant prognostic potential (at log-rank p < 0.05) are shown in Fig. 2c.

Secreted proteins where we identified links to cancer are shown in Supplementary Table 6.

The analyses shown here are in part based upon data generated by the TCGA Research Network: https://www.cancer.gov/tcga.

## Data Records

### Imaging data

All images associated with the screens are deposited in the BioImage Archive (EMBL-EBI) at S-BIAD1013^48^.

### Contents of the dataset

#### All images from the primary screen

Images are separated into two folders – p21 or p27, depending on the CKI immunostained in that repeat. Each of these folders contains 28 folders – one for each plate, labelled by PlateID.

Primary_analysis.xlsx is the analysis of the primary screen data. PlateID corresponds to image labels. Each row represents average data from nuclei in a single well. Column names describe the parameters measured, including: number of nuclei, nuclear area, nuclear roundness, Hoechst intensity, P-Rb intensity, CKI intensity, EdU intensity (all intensities calculated in the nucleus and in the nuclear ring region), EdU ratio (nuclear/nuclear ring intensity of EdU), number of EdU negative cells, number of P-Rb negative cells, percentage EdU negative cells, percentage P-Rb negative cells, percentage EdU negative cells – robust Z-score, and percentage P-Rb negative cells – robust Z-score. Both raw and normalised values are shown for all parameters.

#### All images from the secondary screen

Images are separated by the cell line (MCF7 or HMEC) and plate number.

#### Secondary_screen_analysis

This folder contains both well average and single-cell level analyses from the Hit validation screen (the latter in Secondary_Screen_Analysis_Single_cell_data). Analyses are separated by cell line and plate number. Column names describe the parameters measured, including: number of nuclei, Hoechst intensity, nuclear area, nuclear roundness, Alexa 488 nuclear intensity, Alexa 568 nuclear intensity, Alexa 647 nuclear intensity, Alexa 488 nuclear ring intensity, Alexa 568 nuclear ring intensity, Alexa 647 nuclear ring intensity, EdU ratio, EdU negative cells (fraction), P-Rb negative cells (fraction), percentage EdU negative cells and percentage PRb negative cells.

The analyses shown here are in part based upon data generated by the TCGA Research Network: https://www.cancer.gov/tcga.

## Technical Validation

### Method and control validation

We used PBS as a neutral control (Mock) in our screen and included Palbociclib, a CDK4/6 inhibitor that promotes a robust G0 cell cycle arrest in MCF7 and hTERT-HMEC cells, as a positive control, as well as TGFb1, a secreted protein known to increase the fraction of cells arresting in G0 (Fig. 4a). There are no known secreted proteins or drug treatments that can decrease spontaneous G0 arrest. When validating our controls we also confirmed that DMSO (vehicle for Palbociclib) had no effect on proliferation (Fig. 4b,c).

Both cell lines were treated with each control for 72 hr and received a 24 hr EdU pulse for the final 24 hr of incubation, as described previously. EdU and P-Rb staining (Fig. 4a) were both effective in distinguishing proliferating from non-proliferating cells within a large population (Fig. 4b,c). Quantification of EdU negative (Fig. 4b,c) cells showed Palbociclib significantly increased the G0 fraction to approximately 95%, when compared to neutral controls. Similarly, TGFb1 treatment significantly increased the G0 fraction in a dose-dependent manner, while mock and vehicle controls (PBS and DMSO) induced no change in proliferation.

### Technical replicate consistency

**Fig. 5:**
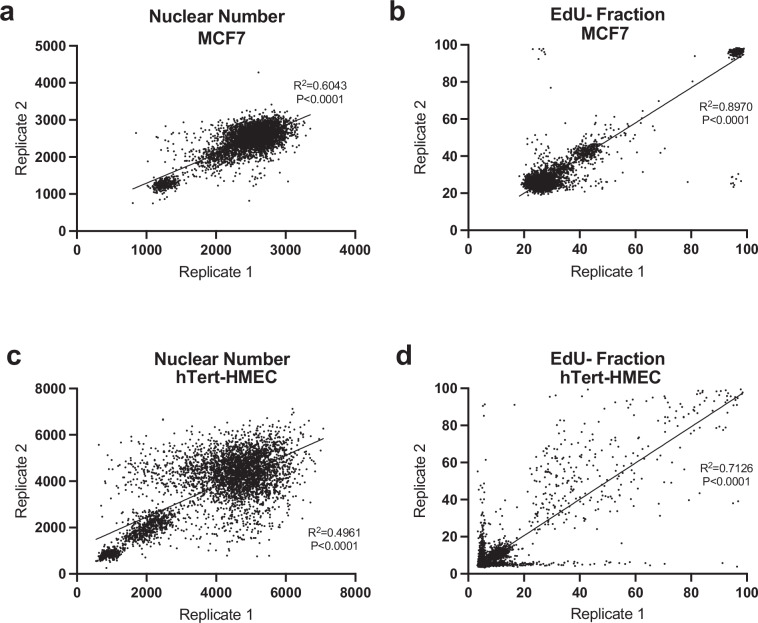
Technical repeats for the secretome screen. Graphs to show the reproducibility of the raw data between the two replica plates for nuclear number (**a**) and EdU negative (EdU-) fraction (**b**) in MCF7 cells (p < 0.0001). Graphs to show the reproducibility of the raw data between the two replica plates for nuclear number (**c**) and EdU negative (EdU-) fraction (**d**) in hTERT-HMEC cells (p < 0.0001). Note that the data for hTERT-HMEC EdU- fraction is noisy for hTERT-HMEC cells but proteins are only called as a hit if they increased the EdU- (G0) fraction in both technical replicates.

We assessed reproducibility of technical replicates for each cell line in the screen using a correlation between replicate values which was analysed with a linear regression model. Specifically, the raw values for cell count and percentage of EdU negative cells of each well were used to calculate correlations between replicates across all plates (Fig. 5). Hits were only called if secreted proteins induced G0 arrest across both replicates.

### Supplementary information

Supplementary Table 1. All raw and normalised image data from the primary screen. Hoechst is nuclear stain. P-Rb labels actively proliferating cells. CKI staining is p21 or p27 (indicated by “Staining” column). EdU labels actively proliferating cells.

Supplementary Table 2. Secreted proteins that promote G0 arrest at both doses of protein from primary screen.

Supplementary Table 3A. Percentage of EdU negative cells in MCF7 across the 21 hits identified in MCF7 cells in the primary screen. 1 refers to the highest dose, 5 to the lowest. Each protein and dose was performed in duplicate wells.

Supplementary Table 3B. Percentage of EdU negative cells in hTERT-HMEC across the 21 hits identified in MCF7 cells in the primary screen. 1 refers to the highest dose, 5 to the lowest. Each protein and dose was performed in duplicate wells.

Supplementary Table 4. Percentage of EdU negative cells in hTERT-HMEC across the 83 hits identified in hTERT-HMEC cells in the primary screen. 1 refers to the highest dose, 5 to the lowest. Each protein and dose was performed in duplicate wells.

Supplementary Table 5. Secreted proteins that promote G0 arrest from secondary screen. N/A is because the protein was not rescreened in that cell line in the secondary screen.

Supplementary Table 6. Secreted proteins that promote G0 arrest and that have links to breast cancer prognosis (excluding all interferons (IFNs) that have well characterised roles in G0 arrest). N/A is because the protein was not rescreened in that cell line in the secondary screen.

## Data Availability

Detailed code describing the assessment of G0 arrest levels across TCGA cancers is available at the following GitHub repository: https://github.com/secrierlab/CancerG0Arrest.
